# Swimlane: Clinical Workflow Redesign in the Nursing Home

**DOI:** 10.1093/geroni/igaf122.2428

**Published:** 2025-12-31

**Authors:** Alyce Ashcraft, Donna Owen, Vickie Ragsdale

**Affiliations:** Texas Tech University Health Sciences Center, Lubbock, Texas, United States; Texas Tech University Health Sciences Center, Lubbock, Texas, United States; Texas Tech University Health Sciences Center, Lubbock, Texas, United States

## Abstract

Workflow redesign is an under utilized skill for understanding how care of older adults (OA) in the nursing home (NH) is conducted. Novel strategies are needed to improve care delivery to the 70% of OAs who would benefit from care in a NH within their lifetime. The aim of this paper is to describe the current workflow and subsequently develop the ideal workflow to improve care delivery in a NH. Data from an IRB approved observational study of NH staff and off-site primary care providers (PCPs) served as the basis for creating the redesign. Event analysis documented baseline existing point of care data collection and communication about a change in resident condition between nurses and off-site PCPs in a single NH. Swimlane and root cause analysis (RCA) methods provided an alternative view of workflow where “at a glance” snapshots were captured then evaluated using a cause-and-effect diagram. Areas for improvement were identified within an “As Is” swimlane diagram that included aspects of training, communication, technology, policies, and staffing. A best case swimlane diagram addressed gaps and areas for improvement showcasing the ideal workflow. The “To Be” swimlane offered solutions to improve workflow such as nurses’ use of critical thinking skills, timely communication with the PCP, and the need to integrate technology into communication. A workflow diagram provides a map and RCA drives potential solutions. Findings suggested a roadmap for research and policy improvement of resident assessment and stronger communication skills between the clinical staff and PCPs.

